# Application of Multispectral Imaging and Portable Spectroscopic Instruments to the Analysis of an Ancient Persian Illuminated Manuscript

**DOI:** 10.3390/s21154998

**Published:** 2021-07-23

**Authors:** Cecilia Rossi, Alfonso Zoleo, Renzo Bertoncello, Moreno Meneghetti, Rita Deiana

**Affiliations:** 1 Department of Cultural Heritage, University of Padova, Piazza Capitaniato 7, 35139 Padova, Italy; cecilia.rossi.2@phd.unipd.it; 2 Department of Chemical Sciences, University of Padova, Via Marzolo 1, 35131 Padova, Italy; alfonso.zoleo@unipd.it (A.Z.); renzo.bertoncello@unipd.it (R.B.); moreno.meneghetti@unipd.it (M.M.); 3 Interdepartmental Center for Research, Study and Conservation of Archaeological, Architectural and Historical-Artistic Heritage—CIBA, University of Padova, Piazza Capitaniato 7, 35139 Padova, Italy

**Keywords:** illuminated manuscript, miniatures, Persian, multispectral imaging, micro-Raman spectroscopy, X-ray fluorescence, fiber optic reflectance spectroscopy, IR-ATR

## Abstract

Illuminated manuscripts are, in general, the final products of a wise and complex interaction of different competencies. In particular, each manuscript reflects uses and techniques rooted in the historical and geographical traditions of the area of realization. Defining the characteristics and the materials in these valuable artefacts is an essential element to reconstruct their history and allow a more precise collocation and a possible comparison with other works in similar periods and areas. Non-invasive methods, mainly using portable instruments, offer undoubtedly good support in these studies. Recent analyses of an ancient Persian illuminated manuscript, combining multispectral imaging and spectroscopic measurements made with portable instruments (XRF, FORS, micro-Raman, IR-ATR) on selected points, provided new data for an improved understanding of this rare book. This study details the possibilities offered by combining these non-invasive methods for an in-depth understanding of the techniques and practices behind the realization of Middle Eastern illuminated manuscripts and provided new perspectives for multidisciplinary approaches to research in this field.

## 1. Introduction

Ancient illuminated manuscripts represent the result of broad, long-lasting collaboration among different competencies. Parchment and papermakers, bookbinders, scribes, and illuminators generally selected materials based on their knowledge [1]. Additionally, differences in traditions, historical contexts, and specific geographic areas often influenced the final product. Not surprisingly, the manuscript was then a precious and rare, generally unique artwork.

The intrinsic complexity that characterizes illuminated manuscripts describes the diversity of aspects that interest these artefacts from an artistic-historical and scientific-technical point of view. Non-invasive, portable techniques, developed and applied for the study of pictorial surfaces at larger scales, offer in this sense excellent support for the characterization and comparison of different manuscripts as well as the various materials used in the same manuscript.

For these studies, the most widely used methods are imaging techniques: multispectral and hyperspectral applications [2,3,4,5,6,7,8,9] and spectroscopic techniques (e.g., XRF, FORS, Raman) using portable and lab equipment [10,11,12,13,14,15,16,17,18,19,20]. In general, most of the scientific studies carried out thus far on manuscripts of different periods and geographical areas have concerned the analysis of the inks [21], the variety and distribution of pigments in the decorations, in some cases, the identification of the techniques of realization, and sporadically, the study of the paper.

Imaging techniques help identify qualitative differences (e.g., transparencies of pigments, underdrawings), whereas spectroscopic methods support defining elemental and molecular properties in single, selected points. The scientific literature shows that Western European manuscripts have been undoubtedly more extensively studied than the Middle Eastern (e.g., Persian, Arabian), only partially analyzed [22,23,24,25,26,27,28,29,30,31,32]. The insufficient literature on these studies is probably due to the greater availability, and the consequent possibility of studying, European manuscripts compared to Oriental ones. In general, scientific analyses are possible and support the study and restoration works of these artefacts. However, the scientific literature reveals a lack of systematic multi-scale approaches based on the combined use of imaging techniques and single-point analyses. In general, multispectral imaging supports identifying underdrawings or pentimenti, whereas single-point analyses facilitate the specific characterization of different materials.

In this case study, we present some aspects of a broader study regarding a 17th century Persian manuscript analysis. In particular, we propose a multi-methodological approach (e.g., multispectral imaging, XRF, FORS, Raman) to demonstrate how the combination of different sensors and techniques can help, in similar contexts, to identify specific details of the decorative apparatus and paper characteristics. The characterization of different mixes and shades of colours, specifically greens, also represents another critical topic in this study. All the results of this multi-methodological study contribute to increasing the overall level of knowledge about this ancient Persian manuscript and, more generally, about Middle Eastern manuscripts still rarely studied in terms of materials and techniques.

## 2. Materials and Methods

**Figure 1:**
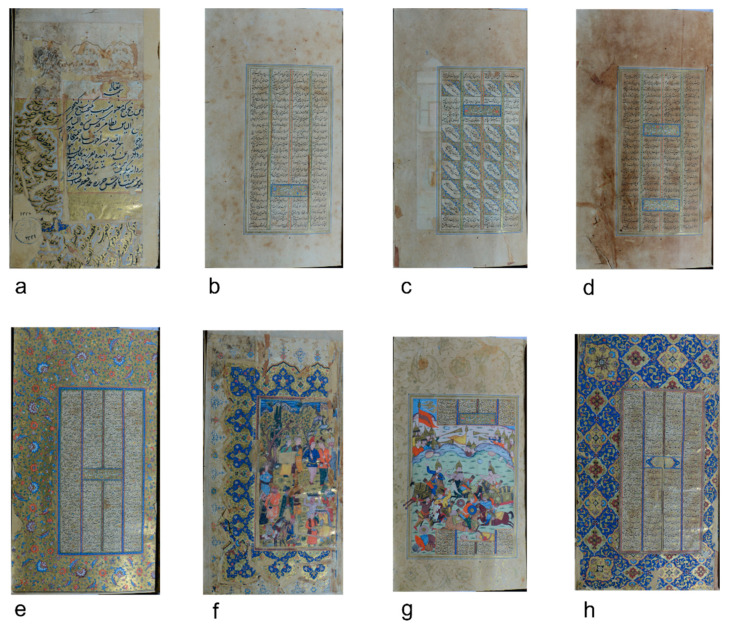
Some examples of different decoration types ((**a**): f. 1 r, (**b**): f. 30r, (**c**): f. 69r, (**d**): f. 37r, (**e**): f. 28r, (**f**): f. 2r, (**g**): f. 53 r, (**h**): f. 3r) in the studied illuminated Persian manuscript M2522_23 (Fondazione—Venice).

The MS2522_23 Quintet (Khamse) of Nizami Ganjavi is a 17th century illuminated Persian manuscript containing 22 full-page miniatures, two diptychs (1 at the beginning and one at the end of the manuscript), many filigree initials, and finely decorated margins and gold/coloured frames between and around the dark text (Figure 1).

This valuable manuscript is a part of the Venetian Fondazione Cini Onlus collection and was analyzed with non-invasive portable methods during its restoration.

In particular, different portable techniques were applied to collect details and information on various pigments and colour blends, identify the characteristics of the paper, develop general information on the artists’ practices, and detect the presence of preparatory drawings or pentimenti.

Following a general to a particular approach, a large dataset of multispectral images covering all the miniatures was planned. This mapping aimed to identify at a general, macroscopic, and qualitative level the main features in the decorative apparatus and the differences among the scenes. Thanks to this preliminary screening, it was possible to identify similar chromatic areas with different spectral responses. In this way, it was possible to choose the specific points of interest to carry out in-depth spectroscopic investigations.

In the context of the analysis of ancient documents and, in particular, in the recovery of details on images, the use of multispectral imaging reveals enormous potential.

Indeed, there are several examples in the recent literature of using this technique in the ultraviolet (UV) and infrared (IR) bands. This method is applied for text retrieval on reuse sheets [4], for the identification of executive details on illuminated documents [9], then for recovering the legibility of text and images on sheets and writing different media that have been blackened for various reasons [5], while also passing through the analysis of specific details on the paper/material used as a support for writing.

A NIKON D800 FR modified reflex camera, 2 NIKON sb-910 flashes, and five specific optical band-cutting filters [6] were used for multispectral analysis.

The removal of the UV and IR band cutter filters applied in the sensor of the reflex cameras restores the complete sensitivity in the range of 300–1000 nm. In this range, the use of filters placed in front of the lens allows one to take pictures of individual bands (UV, VIS, IR), obtaining valuable information on details regarding the state, conditions not visible to the naked eye.

Micro-Raman, fiber optics reflectance spectroscopy (FORS), and X-ray fluorescence (XRF) are generally applied for molecular and elemental characterization of pigments. In particular, in this case, we used two different micro-Raman instruments to analyze selected points in the manuscript. First of all, a portable BWTEK i-Raman Plus instrument equipped with a laser line at 785 nm and a BAC100 probe connected to a microscope were used. Output power was reduced by 50 dB by an external attenuator to avoid any possible damage. A microscope magnification of 4× or 10× was applied. Raman spectra were collected in the range 150–3350 cm^−1^, with 100 accumulations (1 s/acc).

A second micro-Raman spectrometer, Renishaw InVia, equipped with a Leica DM-LM microscope and with laser lines at 514 nm and 633 nm, was also used. Magnification was 20×, with ten accumulations (10 s/acc). For both micro-Raman instruments, the same wavelength range was selected.

A portable μ-XRF Bruker ARTAX 200, equipped with a Mo anode source, adjustable tripod and probe head, spotting areas less than 0.1 mm diameter, was used for XRF analysis. The voltage was set to 45 kV and current to 700 mA; acquisition time was 2 min per spectrum.

FORS acquisitions were carried out with portable equipment consisting of a UV-VIS Xenon lamp connected to a flexible probe encompassing 6 quartz fibers for illumination and a single central quartz fiber to collect the light diffused by the sampled point.

The collected light was fed into an Ocean Optics HR2000+ spectrophotometer through the primary fiber, whose detection range was 300–900 nm. The illumination spot was 3 mm in diameter, acquisition time 40 ms per accumulation, 30 scans, with a boxcar noise filter set to 20.

**Figure 2:**
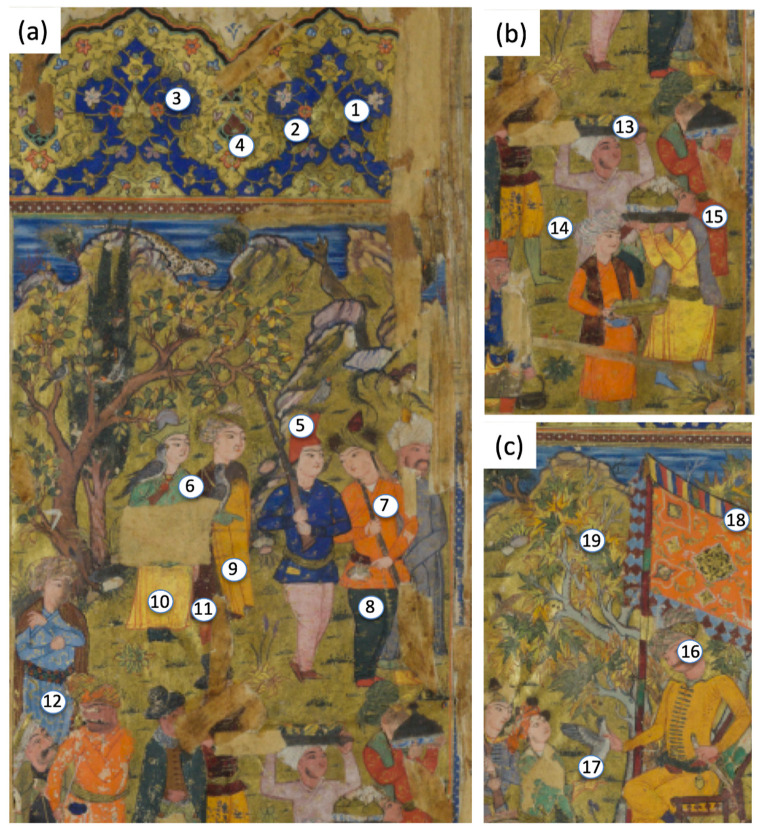
Distribution of point analyses for spectroscopic measurements in folios f. 2r (enlargements (**a**,**b**)) and f. 2v (enlargement (**c**)) of the studied Persian manuscript 2522_23 (Fondazione—Venice).

**Figure 3:**
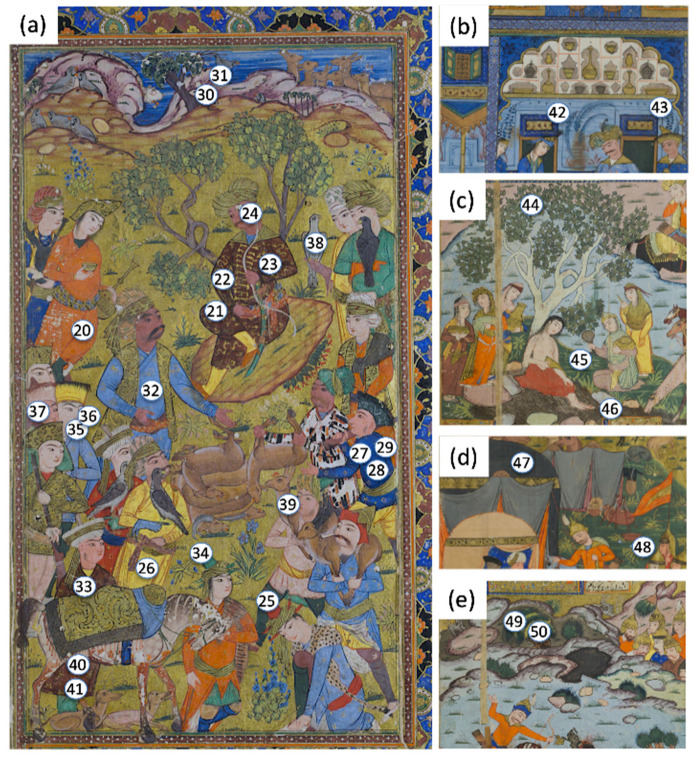
Distribution of point analyses for spectroscopic measurements in folios f. 303v (**a**), f. 180r (**b**), f. 41v (**c**), f. 117v, and (**d**) f. 155v (**e**) of the studied Persian manuscript 2522_23 (Fondazione Cini—Venice).

**Figure 4:**
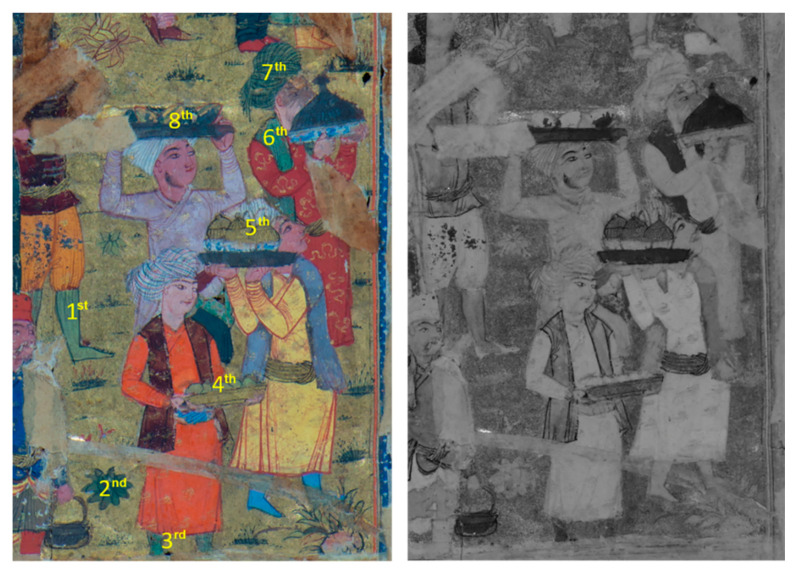
Visible (on the **left**) and IR 850 nm (on the **right**) details of a miniature (folio f. 2r in Figure 2) showing the different transparencies of green colours and mixtures identified in 8 points.

A total of 50 sampling points were collected in spectroscopic analyses, with a broader investigation (19 points) on the first diptych (folios f. 2r–f. 2v), representing the feast of the Sultan (Figure 2). These folios contained a rich palette, covering most of the colours/pigments used in the entire manuscript. The other sampling points were distributed in different miniatures (Figure 3) to characterize blue and green colours, focusing mainly on greens, which appeared quite different, starting from the evidence registered by multispectral imaging (Figure 4).

During the restoration, some folios were temporarily removed, permitting FTIR spectroscopy analysis to characterize the materials. The used range of FTIR spectra was 600–4500 cm^−1^, 32 scans, pseudo-absorbance mode, and ATR correction.

## 3. Results

### 3.1. White Paper Analysis

**Figure 5:**
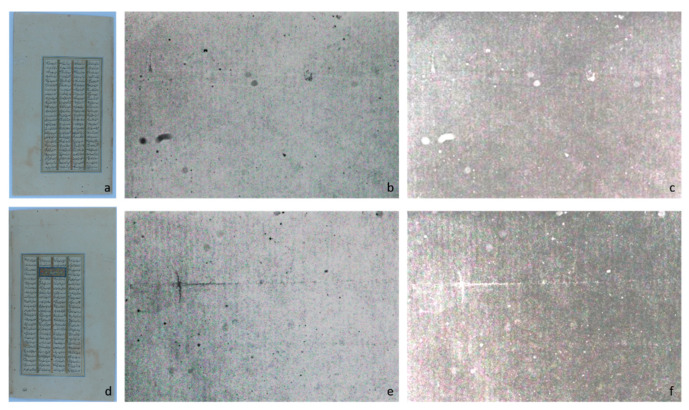
Visible images of the folios f. 284r (**a**) and f. 285v (**d**) and 2 different elaborations of the f. 284r (**b**,**c**) and f. 285v (**e**,**f**) in the 850 nm IR band in the white area of the folios.

**Figure 6:**
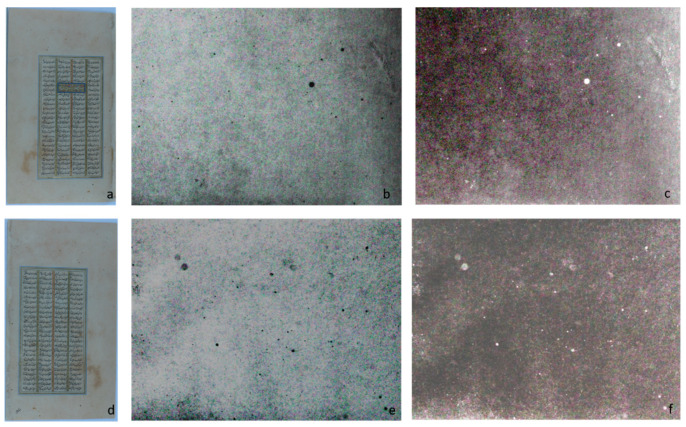
Visible images of the folios f. 285r (**a**) and f. 286v (**d**) and two different elaborations of f. 285r (**b**,**c**) and f. 286v (**e**,**f**) in the 850 nm IR band in the white area of the folios.

Multispectral imaging of white areas in two different folios (f. 285r and f. 285v) enhanced the detection of grooves deriving from the looms used for paper preparation (Figure 5 and Figure 6).

The type and texture of grooves is a relevant codicological parameter to establish the kind of paper, i.e., if the paper was of European or Islamic production [33]. ATR-IR was applied on white areas to characterize the materials used in paper manufacture.

The nature and type of grooves, quite dense and uniform, revealed by the multispectral analysis (Figure 5 and Figure 6), suggest an Islamic production. Additionally, in all the examined folios, no watermarks were identified, indicating the likely Islamic output of the paper.

ATR-IR measurements were carried out on white areas of both removed folios (f. 285r and f. 285v).

**Figure 7:**
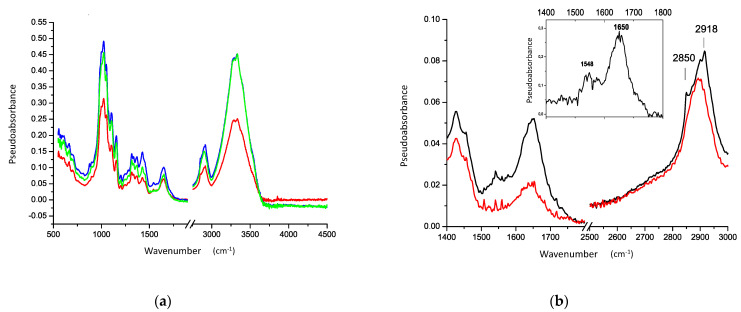
(**a**) FT-IR spectra acquired in correspondence of 3 different points of the white margin in f. 285r; (**b**) Comparison between IR spectrum of the white margin in f. 285r (black spectrum) and the IR spectrum acquired on a pure cellulose sheet (red spectrum). The difference between the black and the red spectra is shown in the window.

For all sampled areas, the IR spectra appeared very similar (Figure 7a) and typical of cellulose, the main component of paper. The strong peak at 1023 cm^−1^, due to C-O stretching mode, the complicated pattern between 1200 and 1400 cm^−1^, and the strong broadband between 3000 and 3500 cm^−1^, due to OH stretching mode, are specific for cellulose. The 1023 peak was structured in distinct, sharp peaks related to the different C-O bonds in cellulose, indicating a good quality paper without degradation. Moreover, the OH band was characterized by small features around 3400 and 3500 cm^−1^, related to slightly different OH groups, suggesting good cellulose crystallinity. In Figure 7b, the ranges 1400–2500 cm^-1^ and 2500–3000 cm^−1^ of the IR spectrum on blank areas of folio 285 (black line) are magnified, as the main variations a paper can show in the IR spectrum because of surface treatments, sizing, or degradation occur in this region. As a reference (in red), the IR spectrum of a pure cellulose paper is shown. The two spectra, red and black, were normalized for the 1023 peak, which generally is contributed only by cellulose. The window shows the spectra difference, obtained by subtracting the red spectrum from the black one (Figure 7b). Two bands with maxima at 1548 and 1650 cm^−1^ are evident, attributable to the amide I and II protein peaks. This outcome indicated a gelatin-based sizing, as typical of western paper, or a shiny coating applied using egg tempera or casein, as usual in eastern paper production.

### 3.2. Details in the Miniatures

**Figure 8:**
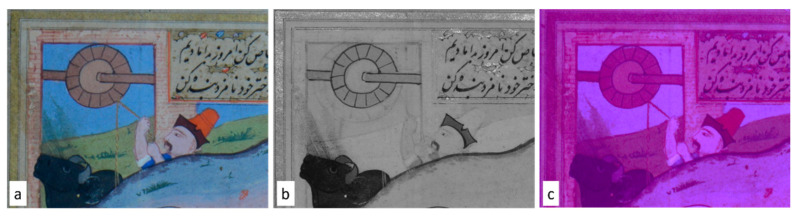
Visible (**a**) IR (**b**) and UV (**c**) folio f. 26r details of the studied Persian manuscript M2522_23 (Fondazione Cini—Venice).

As previously described, in addition to the information obtained on the structure of the paper, supporting the ATR-IR investigations (Figure 5 and Figure 6), multispectral imaging provided information about the presence of different transparencies in pigments, including greens (Figure 4). The multispectral analysis also made it possible to obtain important details on the miniatures and pentimenti presence (Figure 8b).

**Figure 9:**
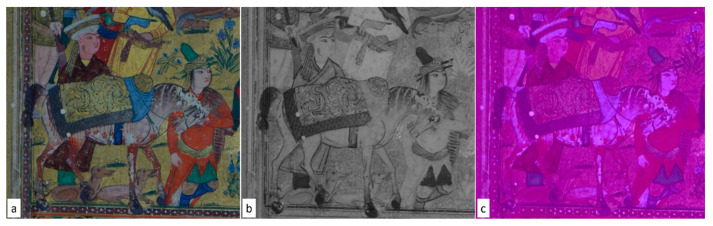
Visible (**a**) IR (**b**) and UV (**c**) folio f. 303r details of the studied Persian manuscript M2522_23 (Fondazione Cini—Venice).

In particular, an interesting aspect, in addition to the transparency of apparently similar colours in the visible range (e.g., greens and blues), was created by the presence of a golden background in the yellow/green scenes (e.g., meadows, texts) or other decorated parts as clearly visible in Figure 8b and Figure 9b.

Gilded pigments were probably applied to all the miniatures for which precious details were foreseen (swords, decorations in the texts, etc.). The golden details were obtained by leaving between corresponding elements a gap that makes golden the only part of interest. In contrast, the remaining parts are covered by other coloured pigments over the background.

### 3.3. Artist Palette

The spectroscopic study carried out over the illuminated manuscripts MS2522-23 concerned a selection of principal pigments, dyes, and pigment mixtures present in the valuable book. Pigment identification was based on the elemental composition derived from XRF analysis and comparison with collected Raman spectra and reference samples. When the reference sample was not available, the literature reference database was adopted [34]. In addition, FORS analysis provided complementary information based on comparisons with online reference spectra databases [19].

The principal palette included the following:

- Lead white (2PbCO_3_.Pb(OH)_2_), easily identified by strong Pb signals in the XRF spectra and by the characteristic Raman peak at 1050 cm^−1^ ca. It was used both as a white pigment and in mixtures with other pigments to obtain lighter hues.
- Minium/red lead (Pb_3_O_4_) for the orange areas, unequivocally characterized by all three spectroscopies (very intense Pb signals in the XRF spectra concerning other areas; sharp inflexion points in the UV-Vis spectra at 571 nm; characteristic Raman peaks, even the weakest, at about 546, 390, 388, 340, 311, e 220 cm^−1^). Red lead was also employed in a mixture, or overlaid, with lead white in the reddish horse coats, as Raman analysis indicated.
- Orpiment (As_2_S_3_), appeared as the preferred pigment employed in yellow areas. It was used alone in the light-yellow details (spectroscopic features: As signals in XRF spectra; specific inflexion points at 480nm of the reflectance spectra; strong characteristic Raman peaks at 380, 352, 308, 290, 200, 177 e 152 cm^−1^). It was mixed with ochres/earth in the darker tones suggested by FORS analysis because the reflectance curve presented the inflexion point at 580 nm and the typical profile of ochres. A similar mixture was presumed for the light brown hue of deer coats, although only Raman analysis was carried out here, and it highlighted the presence of orpiment. However, Raman signals of ochres, often broad and weak, were probably covered by strong orpiment signals. In dark green areas, the artist employed a mixture of orpiment and indigo identified by Raman spectra and confirmed by XRF and FORS analysis (see below in the text for further information).
- Cinnabar/vermilion (HgS) for the bright red tones, clearly characterized by the high amounts of Hg signals in XRF spectra, the typical sigma-profile of reflectance spectrum with an inflexion point at 600 nm and the strong Raman peaks at 340, 281 e 250 cm^−1^. Raman analysis showed that cinnabar was employed in a mixture with red lead for darker skin tones. The same combination, lightened by lead white, was used in the intermediate skin hues.
- Earths/ochres, complex mixtures of Fe(III) oxides, hydroxides, oxo-hydroxides, and silicates and some other transition metal compounds. Red ochres were identified in dark red areas, as indicated by the distinctive inflexion point at 580 nm and the absorbance maximum at higher wavelengths in the FORS spectra, combined with strong iron signals in the XRF spectra. As already mentioned, natural ochres/earth were found in a mixture with other pigments to give, e.g., dark yellow or light brown hues.
- Verdigris (copper acetates), found in aqua-green areas and lapis lazuli, were extensively used in blue areas (their spectral features will be discussed more thoroughly later in the text). In dark blue regions, the presence of organic dyes in a mixture with lapis lazuli was not excluded, but there was no clear spectroscopic evidence. Lapis lazuli was lightened with lead white in light blue areas.
- Silver (now oxidized) was employed to depict the water in the illumination with the princess at the source. The metal was identified by XRF analysis, along with alteration compounds such as chlorides.

The spectroscopic analysis carried out on pink and light purple areas only showed the presence of lead white. The artist probably employed red lake pigments for these tones, but their presence could be inferred only from the absence of elements characterizing the inorganic pigments of the corresponding colour. Organic reds cause in Raman spectra a high fluorescence background, and their constitutive elements were undetectable by XRF. XRF e Raman analysis showed nothing except lead white presence in grey areas, probably used in a mixture with carbon black. Lastly, it is essential to underline the ubiquitous Pb signals in all XRF spectra.

The summaries of the spectroscopic campaign carried out on f. 2r-2v, f. 303v, and on miniatures of Figure 3b–e are reported, respectively, in Table 1, Table 2 and Table 3.

A specific section in Supplementary Materials shows a comparative XRF analysis of the red-yellow areas, supporting the evidence provided by the Raman-XRF-FORS results. XRF (see Figure S1) confirmed that dark red areas were realized using ochres, light red areas with cinnabar, and yellow ones with orpiment. In only small orange areas, we registered red lead. 

### 3.4. Green and Blue Pigments

Multispectral analysis, as previously mentioned, evidenced the occurrence of greens and blues, appearing almost similar in the visible images but partially transparent in the IR ones, indicating the use of different pigments and mixtures for the green/blue tones (Figure 4).

**Figure 10:**
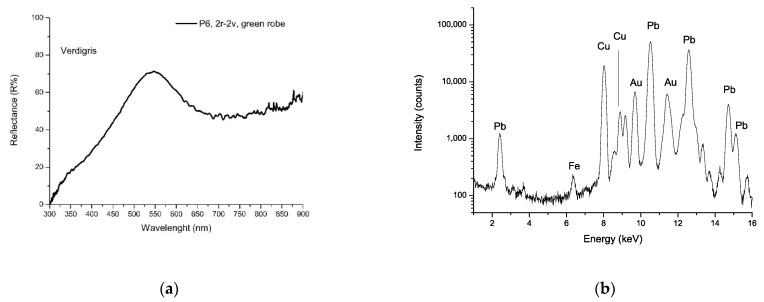
(**a**) FORS spectrum of point 6 in the folios f. 2r–f. 2v, showing verdigris profile. (**b**) XRF spectrum acquired in the same point, showing Cu signals.

Starting from this evidence, specific green points 6, 8, 17, 18, 19, 34, 44, 45, 47, 48, 49, and 50 (Figure 2 and Figure 3) were analyzed. The spectroscopic results (Table 1, Table 2 and Table 3) revealed the occurrence of three types of green tones: (1) blue-green tones in some dress or cap (pt. 6, 34); (2) yellow-green tones in the foliage (the green of leaves, bushes, etc., pt. 19, 44,45,48); and (3) dark green tones (pt. 8, 47) in some dress, fabric, or bush (pt. 8, 47, 49, 50). XRF and FORS analyses unambiguously indicated that the blue-green tone (aqua green) was realized using verdigris, a mixture of different copper acetates. The FORS curve (Figure 10a) showed a typical profile of verdigris, with a relative minimum at 725 nm and maximum at 550 nm, whereas XRF detected a strong copper signal (Figure 10b).

It was impossible to identify the pigment using micro-Raman, due to its fluorescence. This situation is familiar with verdigris, which often induces fluorescent compounds related to degradation reactions occurring in the organic binders. In Persian manuscripts, this fact has been attributed also to the presence of saffron mixed with verdigris, as preservative and dye [32]. An overlooked aspect is the frequent absence of verdigris pigment grains, preventing the detection of the pigment through Raman; this could also be related to verdigris’ propensity to dissolve in proteinaceous binders, with significant alteration of the original structure [35].

**Figure 11:**
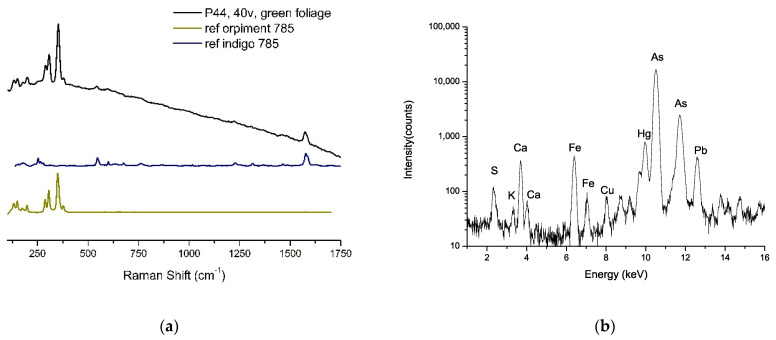
(**a**) Raman spectrum acquired on point 44 in folio f. 40v corresponds to a green area depicting foliage (black line). Reference spectra of orpiment (dark yellow line) and indigo (blue line) are also reported in the figure for comparison. All spectra were acquired with the same laser line (785 nm). (**b**) XRF spectrum of the green bush in folio f. 155v. Strong As signals could be noted. Visible S and Fe signals (and a weak K signal, too) could also be observed.

In the yellow-green or dark green areas (yellow-green for the foliage and grass, dark green for bushes), the illuminators used a mixture of orpiment and indigo, identified clearly by Raman spectroscopy (Figure 11a): peaks observed at 378, 353, 306, 290, 198, 117, 151 and 134 cm^−1^ are typical of orpiment, while peaks at 1581, 1574, 1307, 1248, 1222, 594 e 543 cm^−1^ are characteristic of indigo. XRF confirmed the presence of orpiment, displaying intense signals of As (Figure 11b). This mixture is well-known with vergaut, and its use is documented in various ancient technical handbooks in Western and Eastern areas [36]. Analysis of other Persian illuminated manuscripts often shows the presence of vergaut in the miniatures [28,30].

Only in two points did we detect a significant copper signal using XRF (pt. 17 and 18 in Table 1), but the area was too small for FORS, and Raman was unable to identify the green pigments. According to visual inspection, the green tone in these areas was darker than common verdigris.

**Figure 12:**
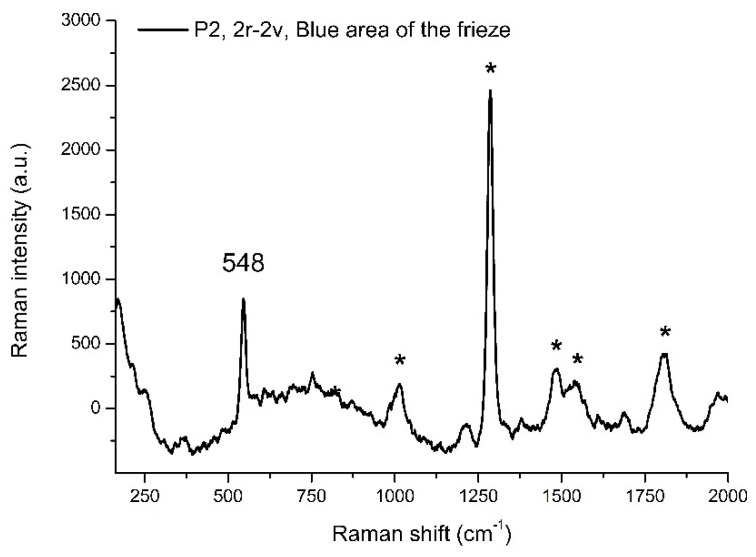
Raman spectra acquired with 785 nm laser line in correspondence with a blue area of the frieze (P2 in folios f. 2r–f. 2v). The distinctive peak of lazurite at 548 cm^−1^ is highlighted, along with the starred satellite peak due to diopside.

A wide range of blue tones could be observed in the miniature; one could distinguish deep blue areas, e.g., in some dresses and the friezes in the opening and closing of the codex, together with many blue shades. A comprehensive characterization of these blue areas was carried out, and in all the examined cases, the pigment was the precious natural ultramarine, i.e., the mineral lazurite, mixed with lead white (pt. 27–29, 32, 42, 43 in Table 2 and Table 3). In the Raman spectrum at 785 nm of the blue frieze (Figure 12), the peak at 548 cm^−1^ is a clear indication of lazurite, but other signals were also visible, including the peak at 1284 cm^−1^ and peaks at 1017, 1483, 1541, and 1815 cm^−1^. A Raman study regarding the characterization of different ultramarine natural and artificial pigments [37] shows that these spot peaks, observed in the Raman at 785 nm of natural ultramarine pigments, can be attributed to diopside fluorescence, a mineral present within the lapis lazuli, and can therefore be used as a marker for the pigment provenance. Finally, as usual in Persian manuscripts, the text was enclosed in gilded frames. Small floral decorations, bordered with red, green, and blue lines, were painted above the gilded frames. A blue copper-based pigment was detected on the blue lines of the frame embedding the text, but it was not possible to acquire Raman or FORS spectra; we can only speculate the use of azurite.

An XRF analysis was also carried out on many green areas (Figure S2 in Supplementary Materials), comparing Cu and As signals as reference for green copper-based pigments and vergaut. The XRF results show that most of the greens in the vegetation were realized with vergaut, while the objects used copper-based pigments.

## 4. Discussion

### 4.1. Folio Characterization

XRF analysis showed an almost ubiquitous occurrence of gold signals on the examined leaves, not only on the illuminated parts but even in white areas. Microscopic examination of the folios indicated small spots of shell gold everywhere on the folios, as suggested by the multispectral imaging, indicating shell gold sprinkling on the leaves as preparation before writing and painting.

ATR-IR and multispectral imaging of the white paper indicated good manufacturing and proteinaceous coating. The limited knowledge of paper manufacture in ancient Islamic and Persian areas made the attribution of sizing/finishing solely through this non-invasive analysis uncertain. This knowledge gap recently prompted a comprehensive analysis of more than 200 samples of paper from Islamic production, employing different techniques [33]. That investigation has shown that many folios (more than half of the total) were coated with a shiny finish (“polishing”) with a proteinaceous substance, which could be casein or egg white, according to the ancient treatises. Of note, in this study, several mock-ups were prepared using Whatman paper coated with egg white, and their IR spectrum, with main peaks at 1533 and 1639 cm^−1^, were very similar to the spectra recorded on leaf 284. Particularly relevant also was the region between 2800 and 3000 cm^−1^ (Figure 7b); strong signals appeared at 2850 and 2918 cm^−1^, which could be attributable to a lipid component, maybe deriving from an egg yolk residue, and therefore supporting the assignment to an egg-based polishing.

### 4.2. Illuminated and Decorated Areas

#### 4.2.1. Artist Palette: General Aspects and Interesting Mixes

The pigment palette identified in the decorative apparatus of MS2522-23 broadly matches the known pigments studied for this specific manuscript [22,29]. The spectroscopic cross-analysis confirmed the unicity and high value of the examined manuscript; a rich set of pigments were detected, including expensive pigments such as lapis lazuli and gold pigments, extensively employed in the decorations. Lead white for pure and bright areas, the extensive use of orpiment and cinnabar/vermilion, verdigris for light green regions, lapis lazuli for blues, minium/red lead for orange areas, organic reds for rendering pink and purple tones, silver for water elements: all in agreement with Persian painting practice.

**Figure 13:**
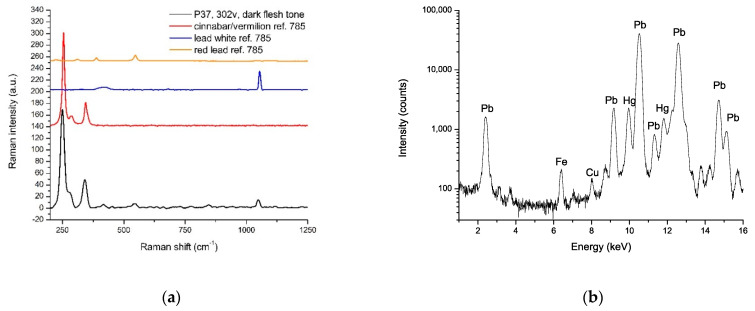
(**a**) Raman spectrum acquired on point 37 in the folio f. 303v corresponding to a dark flash tone (black line). Reference spectra of red lead (orange line), lead white (blue line), and vermilion (red line) are also reported in the figure for comparison. All spectra were acquired with the same laser line (785 nm). (**b**) XRF spectrum of the Sultan flesh tone (P14 in the folios f. 2r–f. 2v). Strong Pb signals and Hg signals could be observed.

Moreover, historical sources and analytical evidence often stress the particular fondness for mixtures shown by Persian illuminators. According to this tendency, artists mostly preferred mixing pigments rather than using them alone. Lead white was widely employed in combinations to lighten or modulate tones. Vergaut was characterized in green areas depicting vegetation. Interestingly, the intensity of the XRF signal of iron acquired in correspondence of three green areas in which spectroscopies identified vergaut was higher than average (Fe is a ubiquitous element, always recorded in traces). This evidence could suggest the possible use of green earth in the mixture. The examined skin tones provided interesting data because these hues often take second place in the palette characterization, and few data are available in the literature. In addition to that, historical sources do not give much information regarding mixing pigments in general for skin hues. Some recipes were handed down by experience, such as mixing lead white and red lead for skin tones [30]. However, the illuminators employed a mixture of lead white, vermilion, and red lead for intermediate and dark skin tones (Figure 13), a mix characterized only by Clark and Mirabaud in Persian illuminated manuscripts [26].

The ubiquitous XRF signal of lead could be due to the presence of a thin preparatory layer of white lead or the white pigment in the mix with several pigments and dyes.

#### 4.2.2. Green and Blue Pigments

Most surprisingly, the only green pigment reported in the ancient treatise was zangar, corresponding to verdigris, for which several recipes were proposed [28]. This aspect contrasts with the experimental findings on Persian illuminated manuscripts, where malachite was found in some areas of the miniatures in many cases, combined with other primary green copper pigments such as atacamite [24,25,27]. Copper pigments are challenging to identify because they often result in degradation, which is related to fluorescent compounds hampering the detection through Raman. XRF can detect copper, but not the way it is bound. Often, FORS is the only technique to identify in a non-invasive and portable way the type of copper pigment. Unfortunately, copper pigments are also prone to chemical transformation, resulting in a variety of different copper compounds, especially the synthetic green ones such as verdigris [38]. Through XRF and FORS, we identified the verdigris present in several zones of the manuscript. Still, copper green pigments (e.g., pt. 17, 18 in Table 1) were detected in small areas through XRF; visually, the color appeared darker than verdigris, but due to the small area, it was not possible to use FORS. However, the most widespread green detected in the manuscript was vergaut, mainly used in vegetation by the illuminators, likely because this mixture allows one to obtain very natural green shades, with tones from yellow to dark green, in the foliage, a superb imitation of proper vegetation. Excluding the blue line in the frame, the only blue pigment detected was natural ultramarine, i.e., lazurite, used to paint both decorations and miniatures. Still, the blue tones could be very different in the miniatures, due to a balanced mixture with white lead, indicating the high level of skill attained by the Persian illuminators.

## 5. Conclusions

This study detailed the results of a thorough investigation of an ancient illuminated Persian manuscript with multispectral imaging and portable spectroscopic measurements. In general, the scientific literature has presented few technical studies on Persian manuscripts compared to European ones. For this study, a holistic approach was adopted, considering that the manuscript had to be viewed as a unicum, where the single parts are strongly related to each other. New data were provided on the artist palette, considering 50 representative points using Raman, XRF, and FORS methods. ATR-IR analysis combined with multispectral imaging of the white folios clarified the nature, size, and types of grooves in the folios and the possible presence of watermarks. Concerning the palette, green and blue areas were deeply characterized, revealing different choices in realizing the hues, with natural ultramarine applied as the only blue pigment.

In contrast, green colors were realized either as a mixture of orpiment and indigo (vergaut) or mainly with verdigris. Different amounts of orpiment and indigo to modulate the green tones in the foliage, from yellow to dark green were revealed. This mix of orpiment and indigo was mirrored in the lighter and darker areas in the IR 850 nm image. Concerning the leaves, ATR-IR investigation supported the presence of a polishing, likely egg-based, and multispectral imaging indicate the occurrence of almost transparent laid lines with no clear watermark. These latter data support the assignment to Islamic paper production.

## Figures and Tables

**Table 1:** Summary of analytical results from spectroscopic methods for points of folios f. 2r–2v indicated in Figure 2. F means FORS spectroscopy, X means XRF spectroscopy, R means Raman spectroscopy with 785 nm laser line, R5 and R6 respectively represent Raman spectroscopy with 514 nm and 633 nm laser lines.

N.	Description	Applied Spectroscopies	Pigment Assignment Based on Cross Data
1	Frieze, orange flower	X	Minium/red lead
2	Frieze, blue area	F, X, R	Lapis lazuli (possibly mixed with an organic dye/lake)
3	Frieze, pink flower	X, R	Lead white probably in mixture with red lake
4	Golden area	X	Gold
5	Red hat	F, X	Cinnabar/vermilion
6	Green robe	F, X	Verdigris
7	Orange robe	F, X	Minium/red lead
8	Green pants	F, X, R	Orpiment and indigo in mixture; lead white in mixture or in the preparatory layer
9	Dark yellow robe	F, X, R	Orpiment (and possibly red ochre in mixture)
10	Light yellow robe	F, X, R	Orpiment
11	Dark red robe	F, X	Red ochre
12	Blue robe	F	Lapis lazuli
13	Green leaf	X, R	Orpiment and indigo in mixture
14	Grass, yellow area	X	Orpiment
15	Purple robe	F, X, R	N.d. (only lead white in mixture or in the preparatory layer)
16	Sultan, flesh tone	F, X, R	Minium/red lead, cinnabar/vermilion and lead white in mixture
17	Green tray	X	Copper-based pigment
18	Flag, green area	X, R6, R5	Copper-based pigment, possibly mixed with an organic dye/lake; cinnabar/vermilion in trace
19	Green foliage	X, R	Orpiment and indigo in mixture; possibly Fe-based pigment

**Table 2:** Summary of analytical results from spectroscopic methods for points of folio f. 303v indicated in Figure 3a. F means FORS spectroscopy, X means XRF spectroscopy, R means Raman spectroscopy with 785 nm laser line, R5 and R6 respectively represent Raman spectroscopy with 514 nm and 633 nm laser lines.

N.	Description	Applied Spectroscopies	Pigment Assignment Based on Cross Data
20	Orange robe	R	Minium/red lead
21 22 23	Sultan red robe	R	Red ochres/red earths
24	Sultan flesh tone	R	Cinnabar/vermilion and lead white in mixture
25	Red hat	R	Cinnabar/vermilion
26	Light yellow robe	R	Orpiment
27 28 29	Dark blue robe	R	Lapis lazuli
30 31	Mounts, purple	R	N.d. (only lead white in mixture or in the preparatory layer)
32	Blue robe	R	Lapis lazuli and lead white in mixture
33	Dark red robe	R6	N.d.
34	Green hat	R	Verdigris (altered?)
35 36	Light flesh tone	R	Lead white in mixture with an undetermined red crystalline pigment
37	Dark flesh tone	R	Cinnabar/vermilion, lead white and red lead/minium in mixture
38	Light gray bird	R	Lead white possibly mixed with carbon black
39	Brown deer	R	Orpiment and natural ochers or earths in mixture
40 41	Brown horse	R	Minium/red lead and lead white in mixture

**Table 3:** Summary of analytical results from spectroscopic methods for points indicated in Figure 3b–e, where the second column shows the specific folios. F means FORS spectroscopy, X means XRF spectroscopy, R means Raman spectroscopy with 785 nm laser line, R5 and R6 respectively represent Raman spectroscopy with 514 nm and 633 nm laser lines.

N.	Illumination	Description	Applied Spectroscopies	Pigment Assignment Based on Cross Data
42	181r	Blu frame	X	Cu-based pigment—possibly azurite
43	181r	Light blue wall	X	Pb-based pigment—lead white and possibly lapis lazuli in mixture
44	40v	Green foliage	X, R	Orpiment (and possibly indigo in mixture)
45	40v	Green grass	X, R	Orpiment and indigo in mixture
46	40v	Brown soil/silver water	X, R	Silver (altered)
47	117v	Green tent	X, R	N.d. (only lead white in mixture or in the preparatory layer)
48	117v	Green grass	X, R	Orpiment and indigo in mixture (and a Fe-based pigment like ochres/earths?)
49	155v	Green bush	X, R	Orpiment (and a Fe-based pigment in mixture?)
50	155v	Light green bush	X, R	Orpiment

## Data Availability

The data supporting this study are available from the corresponding author upon reasonable request.

## Supplementary Materials

The following are available online at https://www.mdpi.com/article/10.3390/s21154998/s1.
