# Supplementary material for: Application of Multispectral Imaging and Portable Spectroscopic Instruments to the Analysis of an Ancient Persian Illuminated Manuscript
Source: Sensors (Basel). 2021 Jul 23;21(15):4998. doi: 10.3390/s21154998 (PMC8348684; doi:10.3390/s21154998)
Supplement: Supplementary file 1 [file sensors-21-04998-s001.zip › sensors-1277446-supplementary.pdf]

Supplementary Materials

Application of Multispectral Imaging and Portable Spectroscopic Instruments to the Analysis of an Ancient Persian Illuminated Manuscript

Cecilia Rossi, Alfonso Zoleo, Renzo Bertoncello, Moreno Meneghetti and Rita Deiana

A comparative XRF analysis of red-yellow areas and green areas was carried out to highlight features of the executive techniques of Eastern painters. Red-yellow areas are prevalent in the manuscript, and four types of pigments were generally detected in these areas: minium (Pb_3O_4), orpiment (As_2S_3), cinnabar/vermillion (HgS), and ochre (Fe_2O_3). By visual inspection, fundamentally, six hues were noticed: orange, red-orange, light red, deep red, light yellow, and deep yellow. Seven points on these areas were acquired: 2 orange, 1 red-orange, 1 light red, 1 deep red, 1 light yellow, and 1 deep yellow.
The relative amount of the different red and yellow pigments in these areas was evaluated by XRF, taking into account the "marker element" for each pigment: Pb for minium; As for orpiment; Hg for cinnabar; and Fe for ochre. XRF counts depend not only on the amount of that metal but also on the atomic number of the element; therefore, heavy metals such as Pb give higher counts than lighter elements such as Fe. However, we were essentially interested in the relative trend for a specific component (e.g., Pb) in the different areas: therefore, we normalized (to [0,1]) the data by dividing the XRF counts for each element by the maximum XRF value found for that element in the data. The normalized data for the seven points are presented in Table S1.

Table S1. Normalized XRF counts for the marker elements of the red-yellow pigments detected in the red-yellow areas. Because each component has different XRF peaks, the main XRF peak was chosen, which is indicated in the table (e.g., K for Fe).

	**Fe K**	**As K**	**Hg L**	**Pb L**
Orange	0,006	0,000	0,002	0,792
Orange	0,004	0,001	0,002	1,000
Red-orange	0,024	0,000	0,172	0,565
Light red	0,009	0,031	1,000	0,062
Deep red	1,000	0,043	0,002	0,186
Light yellow	0,038	0,670	0,003	0,025
Deep yellow	0,013	1,000	0,005	0,145

The plot of the data is shown in Figure S1. The analysis indicated a clear trend: i.e., orange areas were characterized by a higher lead level than the other red or yellow areas. One should be careful when analyzing lead amounts to infer the application of a specific lead-based pigment; lead white was widely used to modulate the tones of other pigments. However, Raman and FORS indicate that minium was used. The XRF comparison in

Figure S1, showing a higher amount of lead in these areas compared to other, similar areas, supports the use of minium. Moreover, the plot shows that the examined red-orange area was realized by mixing minium and cinnabar; this pigment mixture was commonly used to modulate the red tones in Western manuscripts. Pure cinnabar was used for the light-red area, as shown by the high amount of Hg found in this area, but for a deeper red, the painter used a pure ochre, as indicated by the high amount of Fe found. Yellow was realized mainly with orpiment, perhaps using orpiment and a "touch" of minium to obtain a darker tone.

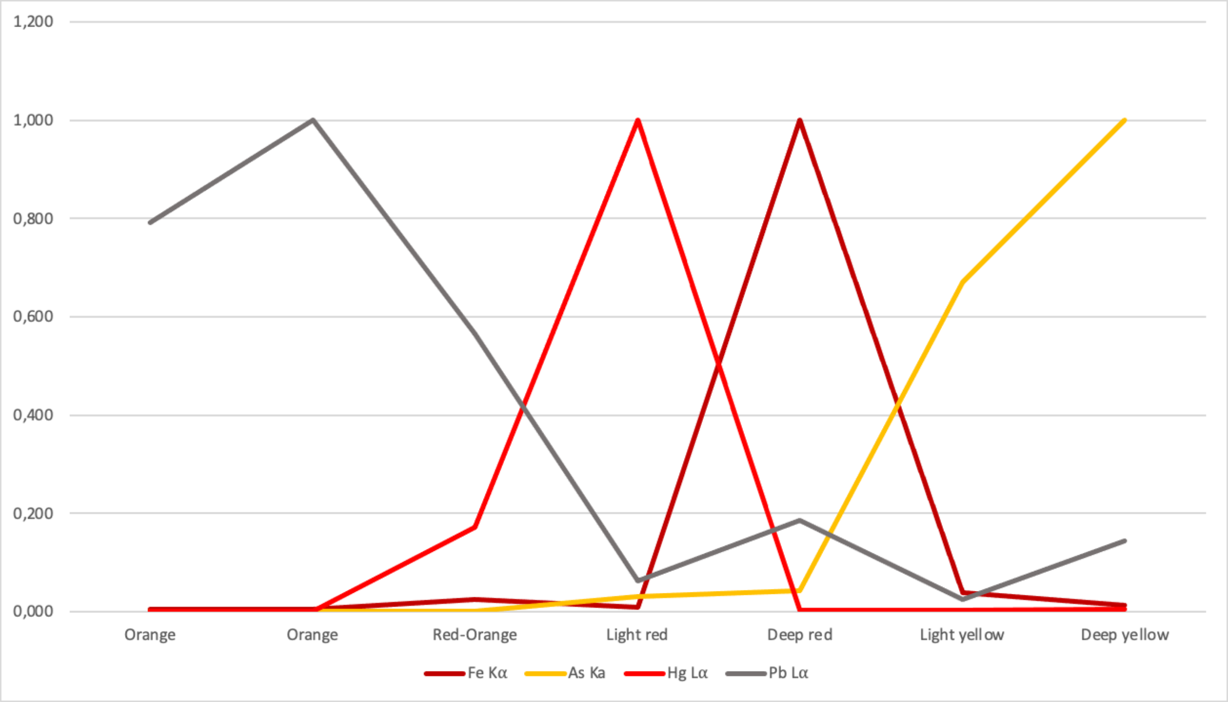

Figure S1. XRF comparative analysis of red-yellow areas

The second XRF comparative analysis was carried out on the green areas. Many Eastern manuscripts are rich landscapes with lush vegetation; the Eastern manuscript painters were masters in depicting such incredible open-air scenes. Because many different hues were present, we evaluated whether correlations were present between the types of painted areas and the applied pigments.

	**Cu K**	**As K**
**Marquee**	0,0054	0,0000
**Flag**	1,0000	0,0171
**Tray**	0,2394	0,0172
**Meadow**	0,0246	0,1413
**Meadow**	0,0059	0,1992
**Meadow**	0,0053	0,3055
**Foliage**	0,0067	0,4065
**Foliage**	0,0213	0,4743
**Moss**	0,0133	0,6969
**Moss**	0,0089	0,8797
**Fruit**	0,0689	1,0000

Table S2. Normalized XRF counts for Cu and As in the green areas.

Through Raman and FORS, we detected that many green areas resulted from a mixture of orpiment and indigo, called "vergaut", but in some small green areas, copper was also present. Figure S2 plots the data presented in Table S2. Two very interesting aspects emerge.

First of all, the examined green areas of objects (marquee, flag, tray, etc.) presented a very low amount of orpiment, with higher values for copper on the small areas, as on the flag and tray, suggesting the application of copper-based green pigments on these small areas. Vegetation (meadows, foliage, moss, fruit, etc.) was vergaut without copper-based pigments. A clear trend was also observed in the relative amount of orpiment; meadows were probably realized using vergaut with a lower amount of orpiment and a higher amount of indigo. By contrast, moss was more "yellow", depicted with higher amounts of orpiment. Foliage green was in-between.

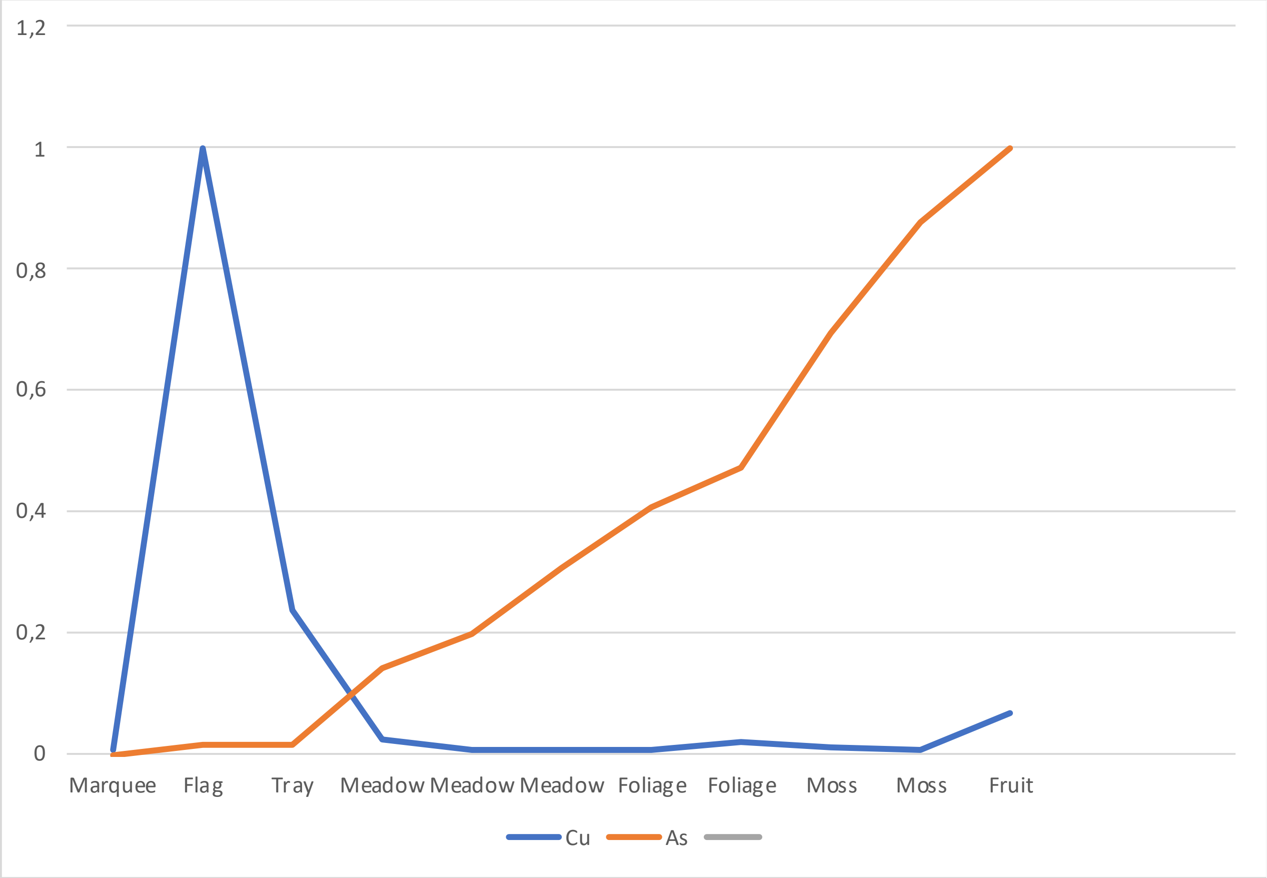

Figure S2. XRF comparative analysis of green areas
